# Using classification and regression tree modelling to investigate response shift patterns in dentine hypersensitivity

**DOI:** 10.1186/s12874-017-0396-3

**Published:** 2017-08-14

**Authors:** Carolina Machuca, Mario V. Vettore, Marta Krasuska, Sarah R. Baker, Peter G. Robinson

**Affiliations:** 1 School of Clinical Dentistry, University of Sheffield, Sheffield, UK; 2 School of Oral and Dental Sciences, University of Bristol, Bristol, UK

## Abstract

**Background:**

Dentine hypersensitivity (DH) affects people’s quality of life (QoL). However changes in the internal meaning of QoL, known as Response shift (RS) may undermine longitudinal assessment of QoL. This study aimed to describe patterns of RS in people with DH using Classification and Regression Trees (CRT) and to explore the convergent validity of CRT with the then-test and ideals approaches.

**Methods:**

Data from an 8-week clinical trial of mouthwashes for dentine hypersensitivity (*n* = 75) using the Dentine Hypersensitivity Experience Questionnaire (DHEQ) as the outcome measure, were analysed. CRT was used to examine 8-week changes in DHEQ total score as a dependent variable with clinical status for DH and each DHEQ subscale score (restrictions, coping, social, emotional and identity) as independent variables. Recalibration was inferred when the clinical change was not consistent with the DHEQ change score using a minimally important difference for DHEQ of 22 points. Reprioritization was inferred by changes in the relative importance of each subscale to the model over time.

**Results:**

Overall, 50.7% of participants experienced a clinical improvement in their DH after treatment and 22.7% experienced an important improvement in their quality of life. Thirty-six per cent shifted their internal standards downward and 14.7% upwards, suggesting recalibration. Reprioritization occurred over time among the social and emotional impacts of DH.

**Conclusions:**

CRT was a useful method to reveal both, the types and nature of RS in people with a mild health condition and demonstrated convergent validity with design based approaches to detect RS.

## Background

Response Shift (RS) refers to changes in quality of life (QoL) independent of health status. It has been defined as a “change in the meaning of one’s self evaluation of QoL as a result of change in the person’s internal standards (recalibration), change in the person’s values of the components of QoL (reprioritization) or redefinition of QoL (reconceptualization)” [1]. These changes may mask or confound treatment effects when QoL is used as an outcome.

Numerous methods have been proposed to assess RS. A common approach to detect recalibration is the then-test [2–6], which adopts a retrospective pre test-post test design. Participants make a retrospective assessment of their health state at baseline based on their current perspective at follow up (‘then’). This approach assumes that the post-test and then-test ratings share the same internal standards, allowing a better estimate of treatment effect than the traditional comparison of baseline and follow up scores. However, this method is prone to bias and lacks standard interpretation [7]. Alternatively, the ideal approach has been used to assess RS with interesting results [8–10]. Participants answer questions about both their actual and their ideal status (e.g. how they would like their QoL ideally to be). Changes in ideal scores at different time points indicate recalibration. This approach is susceptible to ceiling effects if participants consistently regard their ideal as perfection. In addition, ideals may not distinguish between recalibration and reconceptualization [11].

Several statistical methods have successfully detected RS in people with hypertension with coronary artery disease [12], stroke [13], multiple sclerosis [14–16], cancer [17] obstructive pulmonary disease [18]. Structural Equation Modelling (SEM) can measure recalibration, reprioritization and reconceptualization through differences between intercepts or residual variances, values and patterns of common factor loadings respectively [16, 17, 19]. Relative importance measures have assessed response shift in people with inflammatory bowel disease and epilepsy [20, 21]. This method requires longitudinal data on two occasions to detect changes in relative importance weights or ranks of the domains to detect reprioritization. The random forest method has been used as a predictive approach to assess response shift in patients with multiple sclerosis and schizophrenia [22, 23]; this method is an ensemble CRT using bootstrapping of the original dataset.

Classification and Regression Trees (CRT) is a statistical method relative unused in RS detection. CRTs are hierarchical and graphical representations of interactions between variables. Described as flexible and easy to interpret, CRT can supplement traditional analysis to analyse patterns of RS at an individual level even for conditions with a low prevalence [24]. CRT has successfully detected RS among people with AIDS and Multiple Sclerosis. However, these findings have yet to be validated against other methods [25, 26].

RS has not been extensively studied in people with mild health conditions such as dentine hypersensitivity. Dentine Hypersensitivity (DH) is a common condition [27, 28] characterized by short sharp pain in response to an external stimulus [29]. Despite its acute character, repeated episodes of pain over an extended period indicate that DH should be considered a chronic condition [30]. A wide range of prevalence (2.8-98%) of DH has been reported [31–33], but a prevalence of 10% has been accepted as the best estimate of DH around the world [34]. People with DH report more impacts on QoL than the general population, but the condition increases scores in a generic oral health-related QoL measure by less than 10% [35]. Recently, RS was detected in a study nested within a RCT of mouthwashes for DH using the Dentine Hypersensitivity Experience Questionnaire (DHEQ) as a patient reported outcome [9]. Recalibration was detected with both the then-test and the ideals approaches but in opposite directions. The then-test detected an average downward shift in internal standards whereas the ideals indicated an average upward shift. Further investigation could triangulate these results with a statistical approach. Thus, the aims of this study were to describe patterns of response shift patterns in people with DH through CRT and to explore the convergent validity of this technique with the then-test and the ideals approaches.

## Method

### Background in CRT

Classification and Regression Trees (CRT) is found in the literature with different abbreviations (CART, CRT, C&RT, RPART, RTA) depending on the software or the trademark used, but all are based on the method developed by Breiman and colleagues [36]. CRT involves a recursive and iterative procedure widely used in medicine [37, 38], biology [39] and psychology [40]. When compared with other complex modelling techniques, CRT requires the small sample sizes of a minimum of 10 events per variable to obtain a reasonable predictive modelling with stable performance [41].

The technique creates a decision tree using automatic stepwise variable selection to identify mutually exhaustive and exclusive subgroups of a population [36, 42]. The tree acts as a representation with terminal nodes (leaves) representing a cell of the partition, each with a simple model that applies to that cell only. Each node is split through the best variable, maximizing the purity of the resulting nodes; a node is considered ‘pure’ when all the cases have the same value for the dependent variable.

If the primary splitting variable is missing for an individual observation, the data are not discarded but instead, a surrogate variable that has the best similar pattern relative to the outcome variable is used, thereby enabling utilization of incomplete datasets [43]. As a result of the surrogates in splitting the data, the contribution a variable can make to the model is not only determined by primary splits, i.e. a variable can be considered as highly important even when it does not appear as a node splitter. This allows identification of variable masking and nonlinear correlation among attributes [44].

A variable importance score is calculated within the CRT method using the improvement measure attributable to each variable in its role as either a primary or surrogate splitter. The values of all these improvements are summed over each node and totalled. Then, they are scaled relative to the best performing variable; the variable with the highest sum of improvement is scored 100 and all the others will have decreasingly lower scores [45].

To evaluate the reliability of the tree, CRT performs a 10-fold cross-validation. The dataset is divided into 10 randomly selected and roughly equal parts with each part containing a similar distribution of data. The first nine parts of the data (90%) are used to construct the largest possible tree, and the remaining 10% are used to obtain initial estimates of the error rate of the selected sub-tree. The process is repeated 10 times using different combinations of the remaining nine subsets of data and a different 1/10 data subset to test the resulting tree. The results of the 10 tests are then combined to calculate error rates for trees of each possible size and are applied to prune the full tree [46].

CRT is non-model based; it thus allows intuitive interpretations without predefinition of possible interactions among factors and provides a straightforward exploration of non-linear relationships among variables due to its graphical representation [47].

Using Recursive Partitioning and Regression Trees (RPART), Li and Schwartz [26] propose that RS might be inferred qualitatively (interpreting differences in the thresholds, content and order of the independent variables) and operationalized quantitatively as unexpected patterns of contrasting clinical status and self-reported QoL [26]. Following these criteria, this study proposes a definition of RS as changing patterns of DHEQ scores non-coherent with DH clinical status.

### Study design

The study sample was nested within a RCT of mouthwashes for DH [9]. Participants were recruited from the general population as having self-reported DH. The trial was a parallel four-treatment arm: 3 active treatment using desensitising mouthwashes to treat DH and one placebo arm conducted in Hamburg, Germany. All mouthwashes contained sodium fluoride. Ethical approval was obtained from a local independent ethical commission in Freiburg, Germany.

The Dentine Hypersensitivity Experience Questionnaire (DHEQ) was used as a validated outcome measure [48]. The DHEQ has good psychometric properties with high internal reliability (item-total correlations >0.4 and Cronbach’s α=0.86); has demonstrated to be highly responsive to changes in functional and personal experiences of DH in diverse populations [49, 50]. The instrument contains 34 items that record impacts on 5 subscales: functional restrictions, coping, emotions, identity and social impact; items are responded on a 7 point Likert scale with a possible range of 34 to 238. Higher scores represent worse QoL.

Participants were assessed during the trial on five occasions (screening, baseline, week 4, week 6 and week 8) although the current analysis considers only the screening and week 8 assessments. There were two reasons why screening rather than baseline was selected. First, at screening participants underwent an oral examination, completed the DHEQ and started following the study protocol regarding oral hygiene routine. Thus, from the participants’ and clinical perspective, screening is considered as the beginning of the study. Second, the then-test and ideals analysis were conducted with the screening and week-8 assessments to investigate recalibration [9], it is therefore essential to select the same points to perform the CRT analysis and compare the three methods.

The CRT method used the ‘Tree’ command in SPSS, Version 22.0.0.1 (IBM Corp., Chicago, IL, USA) to generate the classification [51].

### CRT model specifications

The analysis was conducted in the active treatment groups (*n*=75). The sample was first classified according to their clinical DH status at week 8 using two measures to assess DH related pain. Positive Dentine Hypersensitivity (DH+) was defined as at least two non-adjacent sensitive teeth with positive tactile (Yeaple probe of ≤ 20g) and evaporative stimuli (Schiff Sensitivity score of ≥ 2). Subsequently, changes in DHEQ scores between screening and week 8 were analysed.

The CRT tree was fitted using and the DHEQ change total score (DHEQ total score _week8_ – DHEQ total score _screening_) as the dependent variable; the clinical status (DH+ or DH-) and the change of the 5 subscales were used as independent variables. These variables were included to reveal different patterns of change in the subscale scores and their influence in the DHEQ total score and additionally to detect changes in subscale order. The analyses were conducted using the following criteria [52]:

- Minimum number of cases in the parent node: 10% of the sample
- Stopping rule for a terminal node: 5% of the sample
- Tenfold cross-validation to validate the tree
- Tree pruning to avoid over fitting with a maximum acceptable difference in risk between the pruned and the sub-tree of 1 standard error
- Missing data handled by surrogate splits

As suggested by Li and Schwartz [26], this study reports the full rather than the pruned tree because in small samples, pruning may omit small groups or participants with subtle changes. Moreover, most studies of RS with CRT have investigated severe conditions. The analysis of small clusters allowed exploration of the relative magnitude of RS in this mild condition.

The interpretation of changes was based on the minimal important difference (MID) defined as the mean change of the total scores in participant`s who reported any improvement in their self-reported QoL. Baker and colleagues [50] reported an MID for the DHEQ of 22 points. This threshold was used as a reference to identify clusters of patients with potential response shift.

### Operationalization of response shift in the CRT model

RS was inferred when the clinical status (Positive or Negative Dentine Hypersensitivity) was inconsistent with the DHEQ score (Table 1). We anticipated that after treatment, participants’ clinical status might improve and they would report less impacts on their QoL, i.e lower DHEQ scores. Recalibration might be inferred when, (i) at follow up, people without clinical DH, reported more impacts on their QoL, i.e they have changed their internal standards upwards or (ii) when at follow up people, with clinical signs of DH, reported lower DHEQ scores indicating downward internal standards. Likewise, reprioritization might be inferred as changes in the relative importance of each subscale to the model over time.

**Table 1:** Operationalization of response shift for DH in the CRT model

Response shift	Operationalization	Qualitative indicator	Interpretation
Recalibration	Changes in subscale scores over time	↓DHEQ scores with worse DH	Downward shift At follow up individuals experience clinical signs of DH but DHEQ total score is lower than at screening
		↑ DHEQ scores with less DH	Upwards shift At follow up individuals experience no clinical signs of DH but DHEQ total score is higher than at screening
		↑ DHEQ scores with worse DH	No recalibration At follow up individuals experience clinical signs of DH and DHEQ total score is higher than at screening
Reprioritization	Changes in the relative importance of each subscale to the model over time		

## Results

### Sample characteristics

Seventy-five participants completed the study at screening and week 8 (Table 2). Their mean age was 37.6 years old (SD=9.8) and 81% were female.

**Table 2:** Sample characteristics active treatment

	Treatment A (*N*= 32)		Treatment B (*N*=26)		Treatment C (*N*=17)		A+B+C (*N*=75)	
	Mean/%	SD	Mean/%	SD	Mean/%	SD	Mean/%	SD
Age	38.6	9.6	34.9	8.6	39.8	11.4	37.6	9.8
Female	78.1		88.5		76.5		81.0	
DHEQ Baseline								
*Restriction*	18.2	6.3	17.2	5.1	18.4	4.4	18.1	5.5
*Coping*	49.4	15.5	48.4	13.7	52.9	13.3	50.3	14.3
*Social*	17.5	6.6	15.8	6.7	18.3	5.8	17.2	6.4
*Emotional*	32.3	6.9	31.8	8.9	31.4	9.9	32.4	6.6
*Identity*	13.7	6.0	11.1	6.0	13.8	8.1	13.9	7.0
Total	131.2	39.8	124.4	34.1	134.8	35.4	129.9	36.5
DHEQ score change *(Post-Pre)*								
*Restriction*	-1.9	4.4	-1.1	5.3	-1.8	5.7	-1.61	4.9
*Coping*	-6.2	14.8	-6.5	13.7	-4.9	10.8	-6.0	13.4
*Social*	-2.7	6.3	-0.9	5.8	-2.4	4.1	-1.0	5.7
*Emotional*	-2.9	8.9	-5.6	8.0	-3.6	8.6	-4.0	8.5
*Identity*	-1.1	6.0	0.2	5.9	-0.6	3.6	-0.5	5.5
Total	-14.8	34.2	-13.8	33.4	-13.4	26.5	-14.1	31.9
Clinical status week 8								
*DH(+)*	46.9		53.8		47.1		49.3	
*DH(-)*	53.1		46.2		52.9		50.7	

The mean evaporative sensitivity scores at screening and week 8 were 2.27 and 1.61 respectively; the mean tactile sensitivity was 12.1 and 25.7 at screening and week 8 respectively. As expected, these values indicated improved DH after treatment. Nonetheless, overall clinical status for DH (i.e. Schiff Sensitivity score of ≥ 2 + Yeaple probe of ≤ 20g) indicates that 49.3% of participants had persistent DH at follow up.

**Fig. 1:**
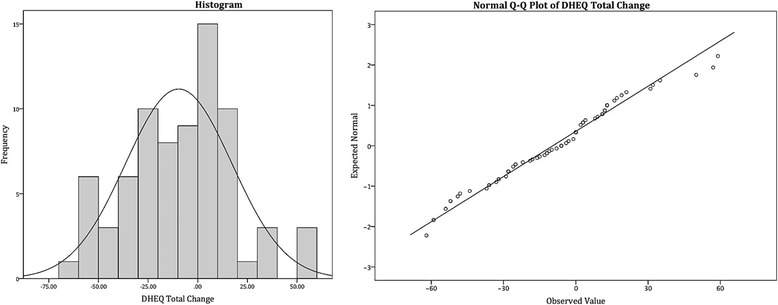
Histogram and Q-Q plot of DHEQ scores distribution

The DHEQ changes scores were compared across the three active treatment groups. Graphic examination of scores distribution was conducted (Fig. 1). The scores were normally distributed (Shaphiro-Wilk’s test, *p*>0.05) and were similar in all 3 groups (one-way ANOVA F(2,72)= 0.14, *p*=0.986; Levene’s test *p*=0.728). In view of this homogeneity the subsequent analyses were performed with the data for the three groups aggregated.

Overall, DHEQ scores decreased by 14.15 points (i.e. less apparent impact at follow up than screening), indicating improved QoL over time.

### Classification tree in the active treatment group

**Fig. 2:**
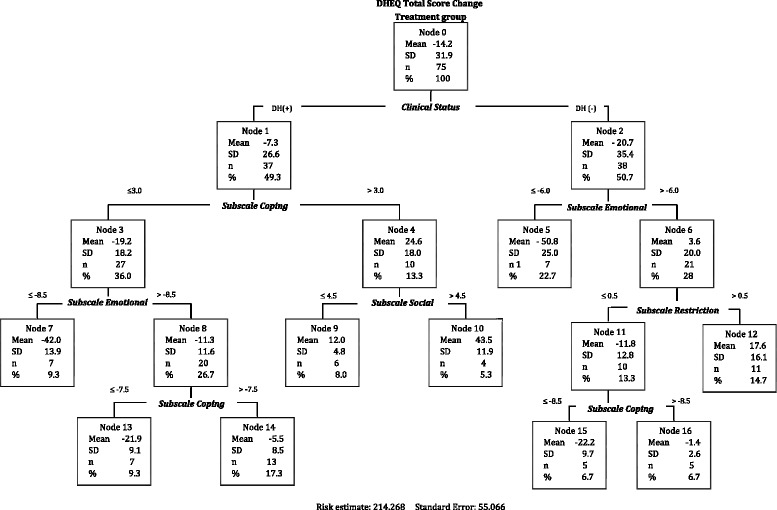
Classification Tree amongst 75 people receiving active treatment for DH

The final tree was developed using 75 valid observed DHEQ changes scores and included the 5 subscales as independent variables ending in 9 terminal nodes (Fig. 2).

#### Model performance

For scale dependent variables (as is the case in this study), the risk estimate is a measure of within-node variance and is used as a criterion of model fit. Lower values indicate a better model. The following equation was applied to calculate model fit [53]:

$$ {{\mathrm{S}}^2}_{\mathrm{e}}=\frac{\mathrm{Risk}\kern0.5em \mathrm{value}}{{{\mathrm{S}}^2}_{\mathrm{y}}} $$

Where,

${S}_e^2$ = Error variance or proportion of variance due to error.

Risk value = Variance within node.

${S}_y^2$= Dependent variable or root node variance or standard deviation of the root node squared.

The proportion of variance due to error is:

$$ {{\mathrm{S}}^2}_{\mathrm{e}}=\frac{214.268}{1018.822}=0.21 $$

The variation in dependent variable explained by the model (S^2^_×_) or explained variance is S^2^_×_ = 1 – S^2^_e_ = 0.79. Thus, 79% of the variation in DHEQ total score was explained by the subscales scores, which had a significant effect in forming the tree, i.e. it is a fairly good model [51].

#### Tree analysis

The first split was for clinical status with 49.3% (node 1) and 50.7% (node 2) of the sample in DH(+) and DH(-) respectively. Both groups reported less DHEQ impact at follow up as reflected in the negative sign of the change mean score. As expected from people with more clinically severe DH (DH(+)), ten participants in the node 4 (13.3%) rated their QoL as worse at follow up.

However, more difference is evident when moving towards the individual level. The terminal nodes represent the best classification for the model. The greatest change was observed in terminal node 7 where the mean change in DHEQ for the 7 participants was -42 points, indicating better QoL at follow up. At the other extreme, node 12 shows that 11 participants rated their QoL as much worse at follow up, represented by 17.6 score points.

#### Possible evidence of response shift

##### Recalibration

According to the operationalization in Table 1, a downward recalibration of internal standards might be manifest as improved QoL in participants with unchanged clinical status. Parent node 3 shows that 36% of participants rated their QoL as better at follow up even though they manifested clinical DH.

Nonetheless, the greatest DHEQ change score in this branch representing downward recalibration might be observed within terminal nodes 7 and 13. Both nodes combined represent 18.6% of the sample with change scores higher than the MID of 22 points.

Upward recalibration might be observed in terminal node 12. Of 75 participants, 14.7% rated their QoL as worse at follow up although their clinical status had resolved, i.e they had shifted their internal standard upwards.

Nodes 5 and 15 represent clusters of participants for whom treatment was effective. With change scores over 22 points these participants’ clinical status and QoL had improved.

##### Reprioritization

**Fig. 3:**
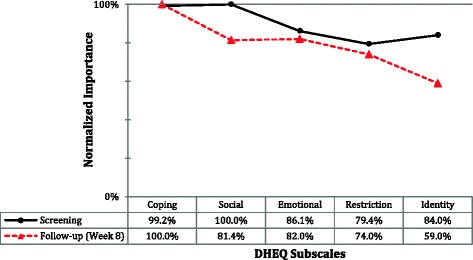
Independent Variable Importance at screening and follow up

The contribution of each independent variable to the model development is termed ‘variable importance’. Reprioritization can be inferred as changes in the order of importance of each subscale from screening to follow up. Figure 3 shows that at screening the social subscale was the most important variable in model development, whereas at follow up the coping subscale was the most important and so on with all subscales.

##### Comparing methods

Both the then-test and ideals rely on questionnaire design to measure recalibration. The then-test uses self-assessment of QoL at baseline (‘pre’) and at follow-up(s) (‘post’), supplemented with a retrospective reassessment (‘then’) of the initial QoL at follow-up(s). In the ideals design, individuals complete the questionnaire twice at both baseline and follow-up, first with regards to how they are at the moment (‘actual’) and second with regards to how they would want things to be ideally (‘ideal’). Arguably, each method uses a different construct of the same instrument. From 75 participants included in the CRT analysis, 43 completed the then-test and 31 the ideals questionnaire at screening and week 8. For the then-test, there was no significant difference between the three active treatment groups as indicated by the one-way ANOVA, F (2, 40)=0.04, *p*=0.96. Likewise for the ideals, there was no significant difference between the three groups (ANOVA, F (2, 28)=1.01, *p*=0.38). As the three treatment groups were similar both for the then-test and the ideals, the comparative analysis was performed for the three treatment groups aggregated.

Table 3 summarizes the magnitude and direction of recalibration as detected by the then-test and ideals using the clinical status as a referent for the three combined treatment groups [9]. For the then-test, the negative sign suggests that people reassessed themselves retrospectively as having better quality of life at baseline than they originally thought (i.e. lowering internal standards). Participants who completed the then-test version of the DHEQ shifted their standards of measurement downwards and were significant for all impact subscales but ‘identity’. In contrast, for the ideals assessment the negative sign for participants indicates that at follow-up they had upward recalibration, i.e on average participants increased their expectations on oral health but this shift was statistically significant only for the emotional aspects.

**Table 3:** Magnitude and direction of recalibration for the then-test and ideals

	N	Mean	SD	t-value	Sig. (2-tailed)^a^
Ideals DHEQ recalibration *(‘Ideal follow-up’ – ‘Ideal baseline’)*	31	-6.19	20.26	-1.70	0.99
Ideals DHEQ subscales recalibration					
*Limitations*		-1.03	3.73	-1.59	0.12
*Coping*		-2.41	7.90	-1.78	0.08
*Social impacts*		-0.76	2.88	-1.55	0.13
*Emotional impacts*		-2.16	5.15	-2.37	< 0.05
*Identity*		0.09	3.65	0.14	0.89
Then-test DHEQ recalibration *(‘Then’ – ‘Pre’)*	43	-15.90	32.32	-3.27	<0.05
Then-test DHEQ subscales					
*Limitations*		-1.70	4.21	-2.69	< 0.05
*Coping*		-6.47	13.55	-3.20	< 0.05
*Social impacts*		-2.51	5.90	-2.86	< 0.05
*Emotional impacts*		-4.18	8.82	-3.15	< 0.05
*Identity*		-1.04	5.79	-1.21	0.23
Total DHEQ score change *(Post-Pre)*	75	-14.14	31.91	-3.83	<0.05

^a^One-sample test

**Fig. 4:**
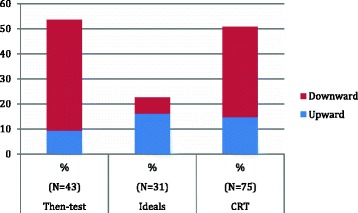
Recalibration for the then-test, ideals and CRT methods

The results of the CRT are comparable with the design-led data (Fig. 4). CRT detected both upward and downward recalibration within the same data. The then-test, detected downward recalibration. With the CRT, downward recalibration can be inferred in participants in terminal nodes 7, 13 and 14 (Fig. 2). The ideals assessment detected overall upward recalibration on the emotional subscale and the CRT detected upward recalibration influenced by emotional changes, as observed in the first split of the tree. Apparently all participants in terminal node 12 (14.7%) experienced recalibration because they did not have clinical DH but showed more impacts in the DHEQ at follow up.

Although the then-test, ideals and CRT show similar patterns of recalibration, this is an exploratory analysis. These methods use a different operationalization of response shift and thus, future research comparing effect sizes using larger samples to evaluate the statistical power of these methods is required.

### Classification tree in the placebo group

**Fig. 5:**
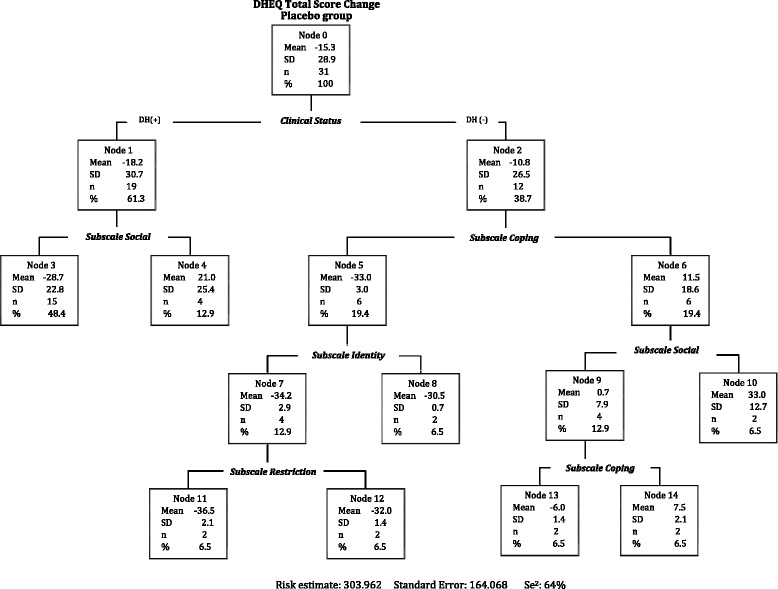
Classification Tree amongst 31 people receiving placebo treatment for DH

A second tree was developed with the placebo group but considering the small sample size this was conducted for illustrative purposes only (Fig. 5). As expected, most participants had clinical sensitivity after treatment (61.3%), but surprisingly, the reported QoL of this group improved more than the treatment group (mean score = -15.32). Furthermore, 48.8% reported an improvement in QoL even though their clinical sensitivity persisted or got worse (node 3). This might be interpreted as participants in the placebo group recalibrating their internal standards downwards after treatment. Due to the small sample, further analysis was not possible in this group.

## Discussion

The first aim of this study was to describe patterns of response shift in people with DH using CRT. The tree analysis suggests patterns of RS consistent with both recalibration and reprioritization. These changes in subjective assessments of QoL might mask treatment effects if this RS is not taken into account when using QoL as an outcome.

Discrepancies between clinical measures and patient-reported outcomes are widely recognised and it may be that RS masks important treatment effects in evaluative research. In this study, 50.7% of participants experienced improved clinical status at follow-up but only one third of people (36%) experienced fewer impacts on their QoL (Fig.1, node 3). Thus, it might be assumed that evaluating treatment effects using simple DHEQ change scores is less responsive if RS is overlooked in this mild health condition. Similar results have been reported previously in dentistry where treatment effectiveness was higher when data analysis considered RS [54]. Kimura et al [55] reported that benefit of dental implants was four times higher when RS was accounted for. Nonetheless, this finding should be interpreted with some caution due to social desirability (i.e., to please the dentist by reporting better outcomes after treatment) and effort justification bias (i.e., underestimation of DH impacts to justify their decision to take part in the study).

Clinical causes and management of DH has been extensively reported [56, 57] but the impact of DH on individuals health cannot be measured by clinical measures alone; incorporation of subjective assessments is essential to determine the effectiveness of treatment strategies of DH [30]. Recalibration of internal standards has been recognized as inherent when using patient-reported outcomes, thus ignoring response shift could lead to invalid conclusions. Response shift should be incorporated in the design of any clinical research involving HRQoL to help clinical investigators and research designers to interpret clinical data effectively.

CRT provided a useful method to analyse patterns of RS. On the left branch of the tree (Fig.1), the first split of node 1 might indicate that people coping with DH reports an improvement in QoL after treatment. But on the right branch, changes in emotional aspects of DH are the most relevant and due to those changes, people rated their QoL as worse after treatment even in the absence of clinical signs of DH (node 6). This might be because after the trial participants were more aware of the impacts of DH on their everyday life; and might rate these emotional aspects as more prominent. However, as the interpretation of changes to identify cluster of patients with RS was based on the MID for the DHEQ of 22 points, it might be that this threshold is not reached due to downward recalibration in some participants. Likewise, in the centre of the tree, social aspects are increasingly important in people, who despite coping with their DH, did not improve after treatment (nodes 9 and 10). According to Schwarz et al [24], CRT allows for the same predictor to have different roles, thus same predictors are repeated across the tree.

Social aspects of DH were the most important variable at screening but at follow-up the coping aspects gained more importance in building the model. Moreover, the social subscale became less important to the model in 19% and the identity aspects were less important after treatment. These findings might be interpreted as reprioritization where DH impacts on different aspects of life over time. Again, this assumption should be interpreted with care as the importance score is specific for each tree. On the one hand, small variations in scores and amounts of data can generate different trees and on the other hand, variable rankings can change considerably comparing trees of different sizes, thus, rankings are strictly relative to a given tree structure [45].

The treatment and placebo trees had similar structure as both showed patterns consistent with downward and upward recalibration (Fig. 3, node 3 and 6 respectively). These findings suggest that recalibration might be a part of the trial placebo effect. Placebo effects found in dentine hypersensitivity [58, 59] have been explained as spontaneous healing or fluctuations of sensitivity [60] as well as response shift. If any therapeutic effect that cannot be explained by the natural course of a condition or any of its pathological mechanisms is attributed to a placebo effect, then response shift might be a type of placebo effect in which patients’ self-assessed health changes are caused by specific psychological mechanisms in the absence of known biological and physiological effects [61, 62].

The second aim of this study was to explore the convergent validity of CRT with the then-test and ideals approaches. The results of this analytic approach are largely compatible with the design-based approaches. Furthermore, CRT offers the additional advantage of observing and explaining complex patterns of RS rather than simply the magnitude. In the original study, the then-test and the ideals revealed recalibration in opposite directions. Importantly, the same results were found in the trees; 36% of participants changed their internal standard downward and 14.7% upward. However, one limitation of this study is that the amount of participants completing both tests was unbalanced (43 completed the then-test and 31 the ideals). Nevertheless, this interpretation is essentially qualitative and the replicability of this model should be confirmed in a different sample.

Nonetheless, these convergent results suggest that the then-test, ideals and CRT measure the same concept. CRT offers the advantage that it is not susceptible to recall bias because it does not require retrospective assessments. In this way the CRT validates the then-test. In addition, many participants shifted their internal standards in the expected direction, i.e. upwards coinciding with the ideals. Another important advantage of CRT is that it does not increase the burden on participants. Unfortunately, with the then-test and the ideals the number of items is doubled at each assessment.

Whilst the CRT method shows promise to detect RS in longitudinal research of mild conditions, its nature is both an advantage and limitation. On the one hand, the graphical representation readily depicts the hierarchy of splits within the sample, but on the other hand the trees have high variance, and slight changes in data might result in different trees.

## Conclusion

CRT appeared to be an effective and efficient research tool to study RS in a mild health condition. It revealed patterns consistent with recalibration and reprioritization in people with DH. To the authors’ knowledge, this report is novel in comparing the convergent validity of the then-test, ideals and CRT as valid methods to assess RS. These findings suggest that response shift might complicate the interpretation of dentine hypersensitivity measures, both clinical and self-reported.

## Abbreviations

CRT: Classification and regression trees
DH: Dentine hypersensitivity
DHEQ: Dentine hypersensitivity experience questionnaire
LTA: Latent trajectory analysis
MID: Minimal important difference
QoL: Quality of life
RPART: Recursive partitioning and regression trees
RS: Response shift
SEM: Structural equation modelling
